# Harmonization of Osteoporosis Guidelines: Paving the Way for Disrupting the Status Quo in Osteoporosis Management in the Asia Pacific

**DOI:** 10.1002/jbmr.4544

**Published:** 2022-04-03

**Authors:** Manju Chandran, Peter R. Ebeling, Paul J Mitchell, Tuan V. Nguyen, the Executive Committee of the Asia Pacific Consortium on Osteoporosis (APCO)

**Affiliations:** ^1^ Osteoporosis and Bone Metabolism Unit Department of Endocrinology, Singapore General Hospital Singapore Singapore; ^2^ Duke–National University of Singapore (NUS) School of Medicine Singapore Singapore; ^3^ Department of Medicine in the School of Clinical Sciences Monash University Clayton VIC Australia; ^4^ Synthesis Medical NZ Limited Auckland New Zealand; ^5^ School of Medicine University of Notre Dame Australia Sydney WA Australia; ^6^ Nuffield Department of Orthopaedics Rheumatology and Musculoskeletal Sciences, University of Oxford Oxford UK; ^7^ Centre for Health Technologies University of Technology Sydney NSW Australia; ^8^ School of Population Health UNSW Medicine, UNSW Sydney NSW Australia

**Keywords:** ASIA PACIFIC, GUIDELINES, OSTEOPOROSIS, STANDARDS OF CARE, TREATMENT GAP, HARMONIZATION

## Abstract

In the Asia Pacific (AP) region, osteoporosis and its consequence of fragility fractures are not widely recognized as a major public health problem. Several challenges including underdiagnosis and undertreatment exist. The Asia Pacific Consortium on Osteoporosis (APCO) is a nonpartisan and apolitical organization comprising musculoskeletal experts and stakeholders from both private and public sectors who have united to develop tangible solutions for these substantive challenges. APCO's vision is to reduce the burden of osteoporosis and fragility fractures in the AP region. Heterogeneity in both scope and recommendations among the available clinical practice guidelines (CPGs) contribute to the large osteoporosis treatment gap in the Asia Pacific. APCO has therefore developed a pan Asia‐Oceania harmonized set of standards of care (The Framework), for the screening, diagnosis, and management of osteoporosis. First, a structured analysis of the 18 extant AP CPGs was completed. Subsequently, a prioritization of themes and agreement on fundamental principles in osteoporosis management were made through a Delphi process of consensus building. This approach, ensuring the opinions of all participating members were equally considered, was especially useful for a geographically diverse group such as APCO. It is hoped that the Framework will serve as a platform upon which new AP national CPGs can be developed and existing ones be revised. APCO is currently embarking on country‐specific engagement plans to embed the Framework in clinical practice in the AP region. This is through partnering with regulatory bodies and national guidelines development authorities, through peer‐to‐peer health care professional education and by conducting path finder audits to benchmark current osteoporosis services against the Framework standards. The principles underpinning the harmonization of guidelines in the AP region can also be utilized in other parts of the world that have similar socioeconomic diversity and heterogeneity of healthcare resources. © 2022 The Authors. *Journal of Bone and Mineral Research* published by Wiley Periodicals LLC on behalf of American Society for Bone and Mineral Research (ASBMR).

## Introduction

Globally, the number of individuals aged 50 years and above at risk for fragility fractures in 2010 was estimated to be 158 million. This number is projected to double by 2040.^(^ ^1^ ^)^ Nowhere is this burden going to be experienced more acutely than in the Asia Pacific (AP), a region of immense physical expanse and topographical heterogeneity, constituted by two continents—Asia and Oceania. Within this vast area that includes East Asia, South Asia, South‐East Asia, and Oceania, and comprising 28 countries and regions, exist populations with diverse, racial, socioeconomic, and cultural norms. The region is home to more than a third of the world's population aged 65 years and over.^(^ ^2^ ^)^ This number is predicted to triple by the year 2050 to reach 1.3 billion.^(^ ^2^ ^)^ Moreover, in parallel with social and economic growth, urbanization is rapidly taking place in almost all the countries in this vast region. This brings with it a significant downside namely sedentary lifestyles. These two critical factors are expected to bring in their wake an exponential increase in fragility fractures.

### Recognizing the problem

The human cost associated with osteoporotic fractures is enormous with hip fractures incurring the greatest morbidity, societal burden, and financial costs.^(^ ^3^ ^)^ It is estimated that in 2018, more than a million hip fractures occurred in China, Taiwan, Hong Kong SAR, India, Japan, Malaysia, Singapore, South Korea, and Thailand. The direct cost of these fractures amounted to USD 7.5 billion.^(^ ^3^ ^)^ By 2050, 50% of global osteoporotic fractures are projected to occur in Asia with an estimated annual incidence of 2.5 million cases, making it the global epicenter of osteoporosis.^(^ ^4^ ^)^ A study from 2011 showed the average age‐standardized incidence rates of hip fractures across the Asia Pacific region to be approximately 300 per 100,000.^(^ ^5^ ^)^ This steep increase in fractures will incur projected costs of almost USD 13 billion.^(^ ^6^ ^)^ However, despite the widespread dissemination of these statistics and the knowledge that implementation of policies to increase case‐finding and treatment rates will result in substantial cost savings,^(^ ^7^, ^8^ ^)^ a very real, ubiquitous, and universal chasm in care exists between those who would benefit from, and those who receive treatment.^(^ ^9^ ^)^

### The challenges

Several challenges in risk assessment, diagnosis, treatment, and prevention of postfracture mortality exist. The unfortunate consequence of the gross underrecognition, underdiagnosis, and undertreatment of osteoporosis even in patients who already have had a fragility fracture is the occurrence of debilitating secondary fractures adding strain to already stretched healthcare systems. In most Asian countries, the burden and consequence of osteoporosis have not received due attention, because of competition with other acute and chronic diseases and it is allocated fewer healthcare resources. Awareness of osteoporosis is markedly low in several countries at both government and healthcare policy decision making as well as amongst healthcare professional and public levels. The International Osteoporosis Foundation (IOF) audit in 2013 revealed that only four countries/regions (Australia, Singapore, Taiwan, and China) officially recognized osteoporosis as an important public health problem.^(^ ^10^ ^)^ Diverse healthcare systems in different stages of development exist in the Asia Pacific and the economies within it span the spectrum from lower middle income (eg, Pakistan, Bangladesh, India) through to upper middle income (eg, Fiji, China) and to high income (eg, Singapore, Australia, S Korea, New Zealand and Japan). The clinical scenarios, fracture risk probabilities, and mortality risk are very different in the former countries compared with the latter. In addition, the availability of treatments and accessibility to healthcare resources vary significantly between these countries and regions. Even within countries, significant differences in epidemiological characteristics of the disease, and in healthcare systems exist. Traditional centuries‐old health practices persist alongside use of the latest medical technologies and pharmaceutical products in almost all the countries in the AP region. Barring Singapore, which is 100% urban, all the other countries in this region have stark urban–rural divisions that accentuate all aspects of the inequities in healthcare resources. In the IOF audit, out of 22 AP countries, 10 had inadequate dual‐energy X‐ray absorptiometry (DXA) resources. Most DXA machines in the region are only available in cities. The underdiagnosis of osteoporosis in rural communities is thus even more pronounced. Lack of reimbursement for bone densitometry testing in several parts of the AP region constitutes an additional barrier to the identification of people with osteoporosis. Similar to that observed in white populations,^(^ ^11^ ^)^ the mortality risk after fracture in Asians is high.^(^ ^12^, ^13^ ^)^ However, although most of the anti‐osteoporosis agents are available in several of the AP countries, reimbursement is highly variable, ranging from 0% to 100% and around 70% of patients with a hip fracture are not treated.^(^ ^14^ ^)^ Osteoporosis is managed by a range of physicians, is not part of the medical curriculum of most AP medical schools, and osteoporosis specialists are generally still scarce in many parts. There is also great variation in terms of how data on fragility fractures are collected and analyzed limiting policymakers' ability to compare performance between AP countries. National reports vary in both the quality and amount of data they capture; eg, regarding inclusion criteria or definitions used. Clinical practice guidelines (CPGs), if available are often not endorsed by the government, not widely disseminated, nor are they updated regularly.^(^ ^10^ ^)^

## A Potential Solution

### APCO and the APCO framework

The Asia Pacific Consortium on Osteoporosis (APCO) is an independent, apolitical, and nonpartisan organization comprised of osteoporosis experts from 19 AP countries and regions.^(^ ^15^ ^)^ It represents many key osteoporosis stakeholders and multiple medical and surgical specialties. APCO has tasked itself to develop tangible solutions to the substantive challenges involving osteoporosis management and fracture prevention in this most populated and fastest growing region of the world.

### The imperative need to harmonize CPGs

When APCO was launched in May 2019, our first step was to derive a shared understanding of the challenges in the diagnosis and management of osteoporosis in member countries, across various specialties. A survey that we executed in 2020 revealed that the AP region had 18 extant CPGs.^(^ ^15^ ^)^ When we analyzed the CPGs, we observed that they were published by different learned bodies, differed in format, and diverged strikingly in the guidance they provided. It was observed that some countries in our region had no guidelines and in others they were out of date; in other countries, guidelines prepared by specialty and subspecialty societies addressed a narrower spectrum of interest limited to the providers in that society. In addition, the same clinical question appeared to elicit different answers by different expert groups and thereby different recommendations.^(^ ^15^ ^)^ The vexing questions that would arise among healthcare practitioners on the ground when faced with these divergent guidelines are self‐evident.

CPGs should ideally enable bridging the gap between high quality evidence, health policy, good clinical practice, and patient preferences. Though indeed they should be adapted to the local context, the heterogeneity in recommendations, and scope provided by the different guidelines on the same subject intrinsically hampers adequate osteoporosis management and serve only to generate confusion among health care providers and policy makers. We realized that to disrupt the status quo in osteoporosis management, a crucial step that needed to be taken was to harmonize the disparate guidelines in the AP region.^(^ ^16^, ^17^, ^18^ ^)^ It was with this goal in mind that APCO embarked on developing a pan Asia Oceania Framework of minimum standards of care for the screening, diagnosis, and management of osteoporosis.^(^ ^19^ ^)^

Previous attempts to develop regionwide osteoporosis guidelines have employed a process of interpretation of available evidence on osteoporosis management by a small, ad hoc working group of experts from within just a few societies/organizations^(^ ^20^ ^)^ or have focused on developing recommendations exclusively for secondary fracture prevention specifically targeted to clinical situations in a particular country, eg the United States.^(^ ^21^ ^)^ Though a few osteoporosis guidelines were reviewed in the process of developing the standards of care for the latter consensus, no systematic analysis of all the individual elements in the guidelines was undertaken. How applicable these consensus recommendations developed in the west are in the AP region is uncertain. It was evident to us during the APCO Framework development that the same guidelines used in countries in the west or even in developed countries in the AP region could not be blindly adopted in their entirety in less developed ones. Neither could the guidance we were developing be prescriptive, authoritarian, or hegemonic. The Framework was therefore conceived in such a way that not only would the standards advocated in it be pragmatic, implementable, and sustainable, but they would be broad, allowing different countries to adapt them to suit local conditions while still adhering to evidence‐based practice.

## The Process of Developing the APCO Framework of Standards of Care

Existing national or regional CPGs were initially identified through a survey among APCO members. Eighteen guidelines from 15 AP countries and regions were thus identified. A list of the guidelines that were analyzed are provided in Table 1. For each guideline, we identified the format in which the recommendations were provided and extracted details of these parameters. A detailed comparative “5IQ” analysis of the content of the 18 individual osteoporosis CPGs was performed. The 5IQ model accounted for the following:

- Identification: A statement of which individuals should be identified
- Investigation: A description of the type of investigations to be undertaken
- Information: A description of the type of information to be provided to an individual
- Intervention: A description of pharmacological interventions and falls prevention
- Integration: A statement on the need for integration between primary and secondary care
- Quality: A description of professional development, audit, and peer‐review activities.

**Table 1:** National and/or Regional Clinical Practice Guidelines Analyzed by APCO

Country or region	Organization(s)	Name of guideline
Australia	Osteoporosis Australia	Osteoporosis prevention, diagnosis and management in postmenopausal women and men over 50 years of age
	The Royal Australian College of General Practitioners	
China	Osteoporosis and Bone Mineral Disease Branch of Chinese Medical Association	Guidelines for the diagnosis and treatment of primary osteoporosis
	Osteoporosis Society of China	2018 China guideline for the diagnosis and treatment of senile osteoporosis
	Association of Gerontology and Geriatrics	
	Osteoporosis Group, Orthopedic Branch, Chinese Medical Association	Guidelines for the diagnosis and treatment of osteoporotic fractures
Hong Kong SAR	The Osteoporosis Society of Hong Kong	OSHK guideline for clinical management of postmenopausal osteoporosis in Hong Kong
Taiwan	Taiwanese Osteoporosis Association	Consensus and guidelines for the prevention and treatment of adult osteoporosis in Taiwan
India	Indian Menopause Society	Clinical practice guidelines on postmenopausal osteoporosis: an executive summary and recommendations
	Indian Society for Bone and Mineral Research	Indian Society for Bone and Mineral Research guidelines 2020
Indonesia	Indonesian Osteoporosis Association (Perhimpunan Osteoporosis Indonesia)	Summary of the Indonesian guidelines for diagnosis and management of osteoporosis
Japan	Japan Osteoporosis Society	Japanese 2015 guidelines for the prevention and treatment of osteoporosis
	The Japanese Society for Bone and Mineral Research Japan Osteoporosis Foundation	
Malaysia	Malaysian Osteoporosis Society	Clinical guidance on management of osteoporosis
	Academy of Medicine	
	Ministry of Health Malaysia	
Myanmar	Myanmar Society of Endocrinology and Metabolism	Myanmar clinical practice guidelines for osteoporosis
New Zealand	Osteoporosis New Zealand	Guidance on the diagnosis and management of osteoporosis in New Zealand
Philippines	Osteoporosis Society of Philippines Foundation	Consensus statements on osteoporosis diagnosis, prevention, and management in the Philippines
	Philippine Orthopedic Association	
Singapore	Agency for Care Effectiveness, Ministry of Health Singapore	Appropriate care guide: osteoporosis identification and management in primary care
South Korea	Korean Society for Bone and Mineral Research	KSBMR Physician's Guide for Osteoporosis
Thailand	Thai Osteoporosis Foundation	Thai Osteoporosis Foundation (TOPF) position statements on management of osteoporosis
Vietnam	Vietnam Rheumatology Association	Guidelines for the diagnosis and treatment of osteoporosis

The 5IQ exercise assessed the extent of consensus or discord when comparing the national CPGs. For example, although all 18 guidelines cited a history of fragility fracture as a risk factor for subsequent fracture, only 13 of them specified the type of fragility fractures and only one of the 18 guidelines advocated benchmarking of Fracture Liaison Services performances against the IOF Capture the Fracture® Best Practice Framework standards.^(^ ^22^ ^)^ There was limited commentary in the guidelines on the need for development of a long‐term management plan and provision of this to either the primary care provider or to the patient, with only the guidelines from Taiwan and New Zealand making specific reference to the need for a long‐term care plan. Similarly, there was limited commentary on audit against standards or continuing professional development related to osteoporosis. A broad range of indications for treatment were cited in the various guidelines, with each guideline typically featuring three or four of a total of 14 indications identified.^(^ ^19^ ^)^

**Fig. 1:**
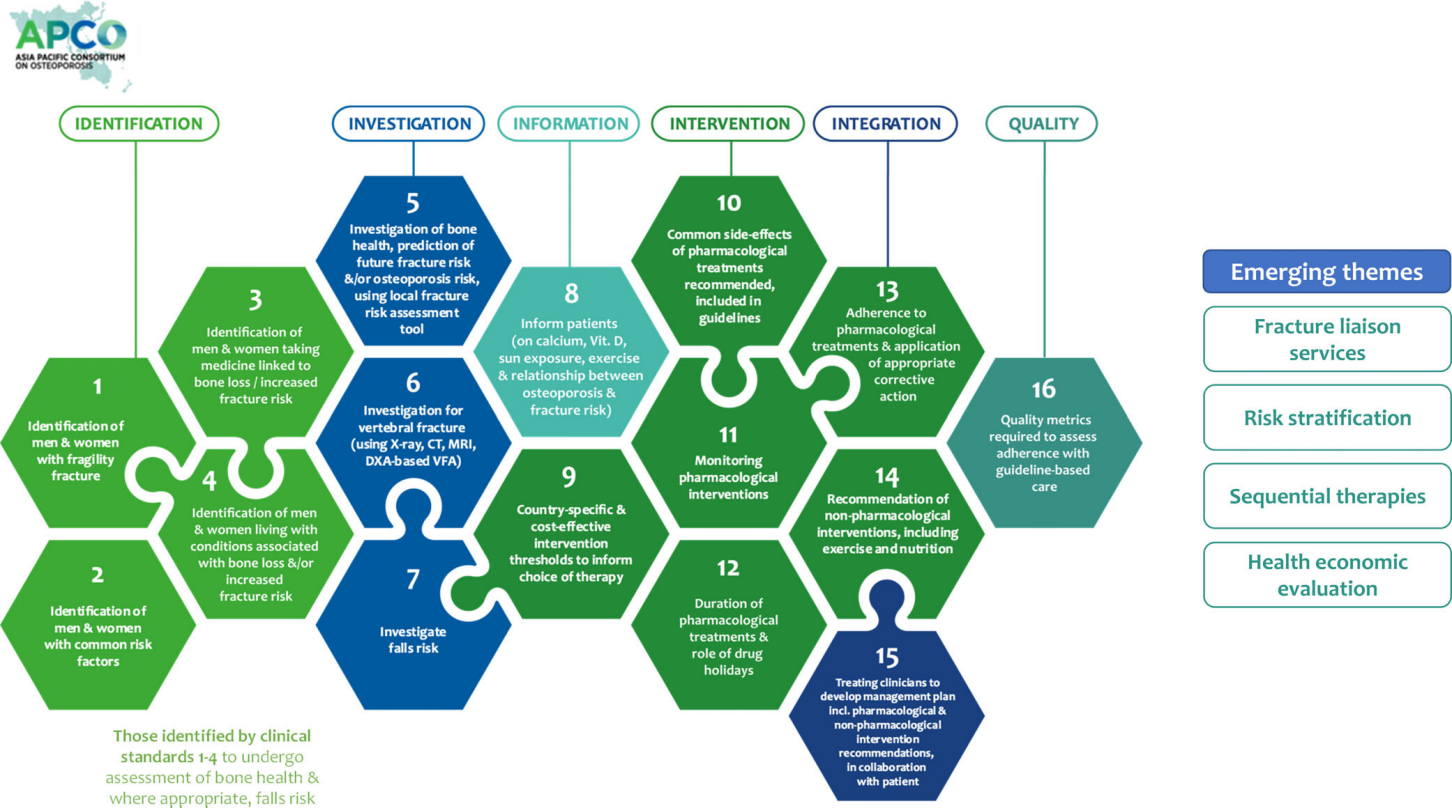
The APCO Framework of standards of care for the screening, diagnosis, and management of osteoporosis.

A four‐round Delphi process in which the then current APCO members participated was subsequently used to develop the standards of care for assessment, diagnosis, and management of osteoporosis for the AP region. For this, APCO members were invited to determine which aspects of care required clinical standards to be developed, based on a list informed by the findings of the 5IQ comparative analysis. The process by which the Framework was developed, including the comprehensive 5IQ analysis and the Delphi process of consensus building has been detailed elsewhere.^(^ ^19^ ^)^ The 16 APCO Clinical Standards of Care are shown as an infographic in Fig. 1 and are described in detail on the APCO website (https://apcobonehealth.org/).

The cardinal principle behind the development of the Framework was that though the standards of care should be pragmatic, it should also provide aspirational guidance to promote best practice. To achieve this, levels of attainment were developed for some of the 16 clinical standards.^(^ ^19^ ^)^ In addition, four recently established and emerging themes in osteoporosis care were identified, namely, the importance of fracture liaison services, the concept of risk stratification, sequential therapies in osteoporosis treatment, and the necessity for health economic analysis to inform indications for specific classes of osteoporosis therapies. The Framework also emphasizes the need for AP countries to develop country‐specific, cost‐effective intervention thresholds for the treatment of osteoporosis, while still adhering to the identified minimum standards of care. It clearly articulates which individuals should be identified for bone health assessment, how they should be investigated, what information should be imparted to them to engage them in their care, which indications for treatment should be advocated, which treatments and other interventions should be recommended for specific patient groups, how integration should occur between primary and secondary care, and what quality metrics should be in place.^(^ ^19^ ^)^

### How can the framework and APCO disrupt the status quo of osteoporosis care in the Asia Pacific?

The blueprint of APCO is to develop plans for optimizing management of osteoporosis and to ensure the harmonization of guidelines across the AP region. To achieve this aim, APCO is working with all the stakeholders in osteoporosis in the different countries to embed the Framework into clinical care. APCO's goal is that all new or revised osteoporosis CPGs in the AP region be aligned with the scope and recommendations proposed in the Framework. To attain this goal, APCO members are making use of opportunities in their individual countries and regions to share the Framework with their colleagues and professional organizations involved in CPGs development. The 16 clinical standards are being distributed in a modular format that should allow easy adoption at the individual healthcare facility, national, or regional level.

A two‐pronged approach is being undertaken. The “bottom‐up” approach involves engaging healthcare providers involved in front line osteoporosis care, providing them with a suite of resources that will enable them to implement the Framework in their individual hospitals, practices, or healthcare clusters, as well as arming them with tools to perform quality improvement projects. The first step in this approach has been through dissemination of healthcare professional peer‐to‐peer educational slide kits developed by APCO (available at www.apcobonehealth.org). This comprehensive “Implementing the minimum clinical standards of the APCO Framework” educational slide kit contains 17 modules, one module for each of the 16 APCO Framework Clinical Standards and one module on recently emerging themes in osteoporosis care. Within the modules are evidence‐based data to help healthcare professionals understand the importance of the clinical standards, epidemiological data, topical guideline summaries, best practice examples, discussion questions, and calls to action. The educational slides have already been extensively disseminated across the AP region and globally. The slide kit has also been accepted by several major universities in the region and is being incorporated into their medical school curricula—a much needed and essential step to rectify the gap in musculoskeletal health education currently prevalent in medical schools. Both the Framework and the educational slide kit are being translated into multiple other languages including Chinese, Korean, and Japanese. It is anticipated that this will broaden the reach and impact of the APCO Framework significantly. The second step in the “bottom‐up approach” is through provision of an APCO Bone health Audit and QI toolkit that is being currently developed and which clinicians across the world can utilize to conduct iterative benchmarking auditing exercises with regard to osteoporosis care in their centres and practices. These exercises can be conducted to establish levels of adherence with those standards of care within the Framework that are amenable to be implemented directly into clinical practice. Once the audits are completed, healthcare professionals and medical centers can embark on implementing changes to their clinical practice. APCO is in the process of engaging QI experts to help train APCO members to conduct such projects in their individual medical centres and practices.

*“Health care is the most difficult, chaotic and complex industry to manage today”* —Peter Drucker

The practice of medicine by clinicians and their capacity and willingness to follow guidelines is not just shaped by clinical knowledge and knowhow, but is also significantly influenced by the healthcare system environment in which they work in. Therefore, even if the Framework of standards of care is disseminated across the AP region, its implementation, and optimal delivery of osteoporosis care may not be realized if the broader environment does not support it. A fine balance exists between clinician autonomy and healthcare regulations. Governmental/institutional health policies and key performance indicators (KPIs) should align, and it is also important to examine the health system through an economic lens, and understand the financial regime and flows within it and how they influence care. If broader system issues and misalignments exist between the various components of the healthcare delivery pathway, clinicians will be unable to provide high‐quality osteoporosis care. To reach an equipoise in this lethal equation, it is imperative that a shift in thinking must occur. To achieve this aim, APCO through its second strategy, the “top‐down approach” is in the provisional stages of engaging policymakers, payors, and guidelines‐development authorities in individual countries in the AP region to broker a shared vision of best practice between them and healthcare providers on the ground.

**Fig. 2:**
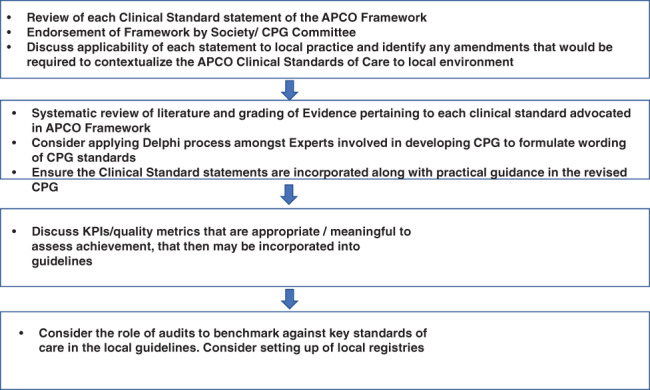
How national osteoporosis societies and CPGs development authorities can use the APCO framework to develop or update CPGs. CPG = clinical practice guideline.

We are fully cognizant that APCO is a consultative body comprised of individual experts and representatives from clinical and corporate fields and not‐for ‐profit organizations and that we cannot be prescriptive nor be perceived as interfering in health policies or political agendas of individual or collective governments. However, we are developing bespoke, country specific engagement plans for each country that the individual APCO member(s) will then implement. A survey of APCO members to identify the societies and specialty colleges that publish CPGs in their respective countries, the specialties involved in the production of these guidelines, the processes and timelines for reviews and updates of existing guidelines, the relevant policy makers in the individual countries, the bodies or organizations that hold healthcare providers to account for delivery of care or incentivize specific outcomes, the regulators of the pharmaceutical industry and healthcare profession, and healthcare funders in the individual countries, etc. has been conducted. An infographic on how national osteoporosis societies and CPGs development authorities can use the APCO Framework to develop new or revise existing national/local osteoporosis guidelines is provided in Fig. 2.

### Policy level changes needed

An action plan is being launched based on the findings we obtained from this survey that will harness members from each country represented in APCO to specifically call upon their governments, parliaments, payers, regulatory bodies, learned societies, healthcare systems, and industry to implement one or more of the following steps to improve osteoporosis care and to mitigate the gaps in its management.

1. Adopt a life course approach to optimal bone health with bone health to be included as a specific target in national chronic disease prevention policies.
2. Integrate osteoporosis, fragility fractures, and falls prevention into national strategies and plans for health; eg, those which aim to address chronic diseases, women's health, healthy aging, long‐term care, and workforce productivity so that resources can be shared.
3. Derive consensus on systematically identifying individuals with osteoporosis and high fracture risk. Decisions on screening should be based on country‐specific epidemiological and economic data. Consider integrating osteoporosis screening into other large‐scale screening programmes; eg, breast cancer.
4. Encourage the implementation of evidence‐based CPGs for osteoporosis prevention and management adapted to the local environment.
5. Ensure reimbursement decisions for diagnostic tools such as DXA scanning, and integrated fracture care reflect the true costs of fragility fractures to the wider healthcare system and society and not consider costs in isolation.
6. Develop nationwide fracture registries to enable monitoring and surveillance of patient health outcomes. Policy makers should also consider how this data can be used to incentivize improvements in quality of care.
7. Actively support education efforts to improve public awareness of osteoporosis, fragility fractures, and falls prevention.
8. Encourage formation of of alliances between different stakeholders including policy makers, health professional societies, the private sector and nongovernmental organizations that are critical to develop and communicate a unified national call for policy change.
9. Encourage creation and adoption of quality metrics against which care can be benchmarked; eg, assessment and treatment of patients at high risk.
10. Facilitate formation of multidisciplinary teams and provision of chronic disease management programmes and pathways.
11. Encourage development and use of fracture risk assessment tools such as Fracture Risk Assessment Tool (FRAX®) that can be used without the need for DXA bone mineral density (BMD) in resource‐strapped or rural settings.
12. Implement high‐quality, sustainable, practices that will ensure development of fracture liaison services and orthogeriatric services that perform according to nationally and internationally recognized standards.
13. Provide high‐quality, accredited education for medical, nursing, and allied healthcare students, healthcare professionals, policy makers, and the public to help raise awareness of the importance of good bone health and fragility fracture prevention.
14. Encourage the pharmaceutical and health technology industry to work collaboratively with professional societies, government organizations, universities, insurance companies, and healthcare systems to develop new treatments and technologies intended to improve patient outcomes.
15. Healthcare systems and research funding bodies to provide resources for research on best practices for care of osteoporosis and fragility fractures.

These steps are ambitious, and it may be difficult to institute all of them in any one country. It might be thus necessary for stakeholders to prioritize and adapt the proposed strategies to the local environment before their implementation.

### The role of collaboration

Collaboration with other regional and international bodies and organizations involved in osteoporosis and fragility fracture advocacy and care has been instrumental in the work of APCO. Almost all the members of APCO are committed and highly involved members of other well‐established professional and scientific organizations and this has brought key expertise to APCO to fulfill its mission. Acknowledging the importance of the APCO Framework, several organizations such as the International Osteoporosis Foundation (IOF), the Fragility Fracture Network (FFN), the American Society of Bone and Mineral Research (ASBMR), the Asian Federation of Osteoporosis Societies (AFOS), and the Asia Pacific Fragility Fracture Alliance (APFFA) have already endorsed it giving it further credibility and cementing its value in the armamentarium of osteoporosis related healthcare resources available globally.

APCO currently has 46 members from 19 countries and regions in the Asia Pacific. This represents more than two‐thirds of the AP region. This itself is a significant accomplishment given that APCO is a small, nascent organization. Obtaining engagement from all the countries and regions that form this vast and heterogenous region is likely impossible. An expanded membership drive exercise is under way and the collaboration with the aforementioned large organizations and healthcare media will enable as wide a dissemination of the Framework and APCO activities as is possible in the Asia Pacific. The APCO Framework launch garnered significant world‐wide attention from more than 350 news and media outlets including organizational media such as that of the IOF and American Society for Bone and Mineral Research (ASBMR), and region wide finance, healthcare, and news wires broadcasting the launch. This broad attention that the Framework acquired reflects the growing interest worldwide in noncommunicable diseases such as osteoporosis and the awareness of the importance of such alliances and collaborations.

Understanding the experiences of patients who live with the devastating disease that is osteoporosis and involving them and their carers in research agendas so that previously unidentified important perspectives can be considered, is critical to improve osteoporosis care. APCO is developing digital patient education platforms and its members include individuals who hold leadership positions in prominent osteoporosis organizations such as the IOF, Healthy Bones Australia, Osteoporosis Awareness Society of Kuala Lumpur and Selangor, and the Osteoporosis Society Singapore who represent the interests of patients. A limitation that we acknowledge is that we do not currently have any osteoporosis patient representative in our membership. We are planning on inviting patient representatives to join APCO in the near future.

## Conclusion

There are currently many challenges in the management of patients with osteoporosis. Implementation of the nonprescriptive, minimum clinical standards proposed by the APCO Framework in new osteoporosis CPGs, or revision of existing ones in the AP region informed by it will support new clinical improvement initiatives and help pave the way for a more holistic approach to osteoporosis care. Although complete harmonization of CPGs across the AP region is unlikely to be ever achieved, the APCO Framework will enable much greater consistency among the national and regional CPGs than what currently exists in this region. Several AP countries that are currently developing new osteoporosis guidelines or are revising existing ones have now agreed to incorporate the clinical care standards from the APCO Framework into the process. We also hope that the core principles that underpin the development of the Framework can reverberate outside the AP region and will be translated into a global groundswell. The value impact of replicating the APCO approach namely harmonizing guidelines across two continents—Asia and Oceania, merits exploration in other regions of the world that have similar socioeconomic diversity and heterogeneity of healthcare resources, including North and Latin America, the Middle East, and Europe. The lessons learned from our odyssey in developing and implementing a dual‐continental set of clinical care standards can be shared with these regions to make a difference to the fragile world in which patients with osteoporosis live.

## Author Contributions

**Manju Chandran**: Conceptualization, Writing of draft, reviewing and editing. **Peter R. Ebeling:** Writing – reviewing and editing. **Paul J Mitchell:** Conceptualization; data curation; methodology. **Tuan V. Nguyen:** Writing – reviewing and editing.

## Conflicts of Interest

MC has received honoraria for speaking engagements and for chairing advisory boards for Diethelm Keller Siber Hegner and Amgen. PRE has received research funding from Amgen, Eli‐Lilly, Novartis, and Alexion and has received honoraria from Amgen and Alexion. PJM has undertaken consultancy for governments, national and international osteoporosis societies, healthcare professional organizations, and private sector companies relating to systematic approaches to fragility fracture care and prevention since 2005; he served as a paid consultant to APCO from September 2019 to August 2020 and now contributes pro bono. TVN has received grant support from Amgen and honoraria for lectures and conference engagements from Novartis, Merck, Sharp & Dohme, Amgen, and Bridge Health Care.

### Peer Review

The peer review history for this article is available at https://publons.com/publon/10.1002/jbmr.4544.

## Data Availability

Data sharing not applicable to this article as no datasets were generated or analysed during the current study
